# Supplementary material for: Structural basis for potent neutralization of human respirovirus type 3 by protective single-domain camelid antibodies
Source: Nat Commun. 2024 Jun 27;15:5458. doi: 10.1038/s41467-024-49757-1 (PMC11211449; doi:10.1038/s41467-024-49757-1)
Supplement: Supplementary file 1 — Supplementary Information [file 41467_2024_49757_MOESM1_ESM.pdf]

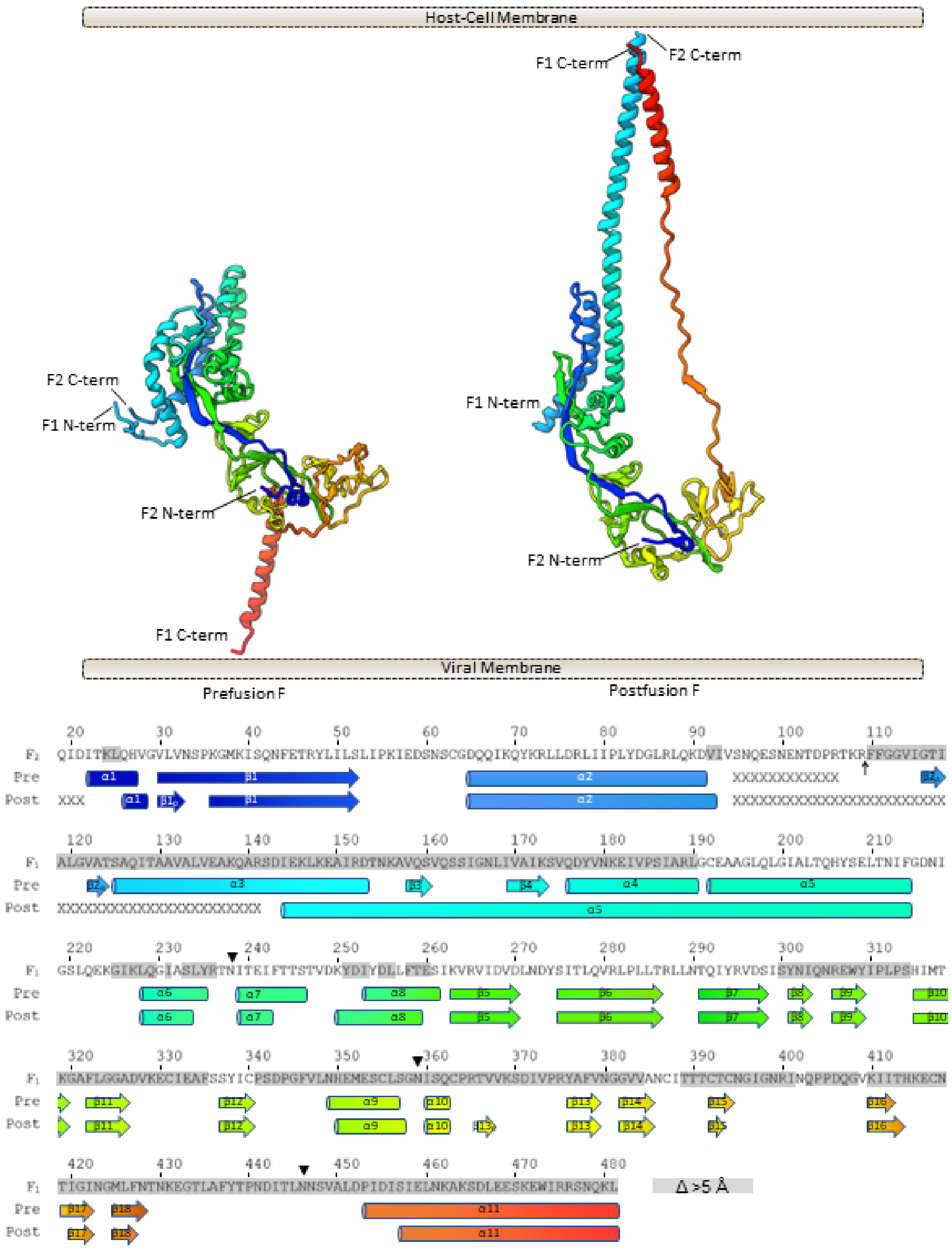

Supplementary Fig. 1: RV3 F pre- and postfusion structure comparison.
(top) Ribbon representation of prefusion and postfusion RV3 protomers, colored as a rainbow from blue to red, N-terminus of F2 to C-terminus of F1, respectively. (bottom) Secondary structures are shown below the sequence, with α-helices and β-strands numbered and shown as cylinders and arrows, respectively. Disordered residues are indicated by an “X”. Sites of N-linked glycosylation are indicated by black triangles, and the F1/F2 cleavage site is indicated by an arrow. Residues that move more than 5Å in the transition between the prefusion and postfusion conformations are highlighted in gray.

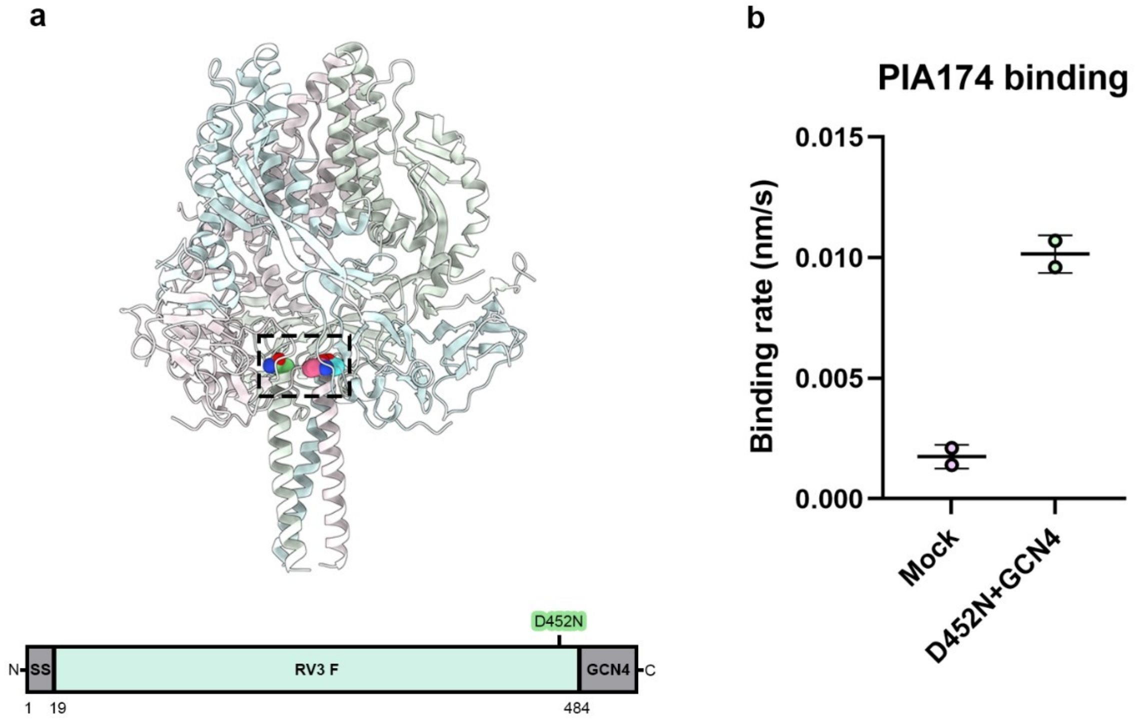

Supplementary Fig. 2: Recombinant RV3 preF antigen used for immunization, binding experiments, and cryo-EM structure determination. a (top) Structure of RV3 preF, shown as ribbon cartoons with each protomer colored light pink, blue, or green. Stabilizing substitution D452N is shown as spheres, colored a darker shade of the corresponding protomer color. (bottom) The gene schematic describes the expression construct, including the N-terminal signal sequence (SS), the RV3 F gene, the position of the D452N substitution, and the C-terminal GCN4 trimerization motif. **b** Binding of supernatant from cells transfected with RV3 preF (D425N+GCN4) plasmid to preF-specific antibody PIA174, measured by BLI. Mock indicates binding of untransfected supernatant. Binding rates were determined using the initial linear slope of the binding curve. Error bars indicate mean values with standard deviation (n=2).

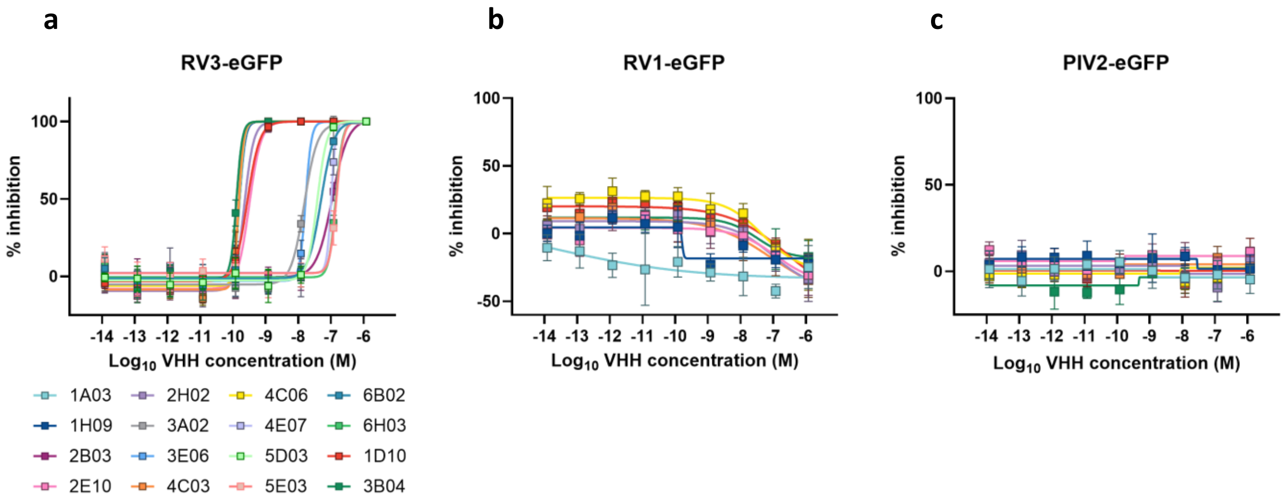

Supplementary Fig. 3: Neutralization of recombinant respirovirus 3 (RV3), respirovirus 1 (RV1), and orthorubulavirus 2 (PIV2) by VHHs. a Representative VHH neutralization curves for recombinant RV3-eGFP, related to Fig. 2a. Calculated IC_{50} values are reported in Supplementary Table 1. **b** Representative VHH neutralization curves for recombinant RV1-eGFP. **c** Representative VHH neutralization curves for recombinant PIV2-eGFP. Source data are provided within the Source Data file.

a

		1st VHH				
		1A03	2H02	4C06	1D10	1H09
2nd VHH	1A03	0.05	0.96	1.03	1.29	1.08
	2E10	0.06	0.72	0.84	0.91	0.79
	4C03	0.04	0.91	1.03	1.10	1.04
	2H02	0.93	0.11	0.00	0.85	0.00
	4C06	1.18	0.03	0.11	0.74	0.05
	6H03	1.13	0.00	0.13	0.72	0.10
	1D10	1.35	0.99	0.95	0.10	0.17
	3B04	1.57	1.05	1.27	0.07	0.17
	3A02	1.35	0.80	1.13	0.00	0.09
	3E06	1.38	0.78	0.90	0.00	0.05
	4E07	0.80	0.63	0.59	0.00	0.00
	2B03	1.25	0.51	0.73	0.01	0.63
	1H09	0.96	0.01	0.08	0.05	0.14
	Buffer	0.00	0.06	0.11	0.11	0.06

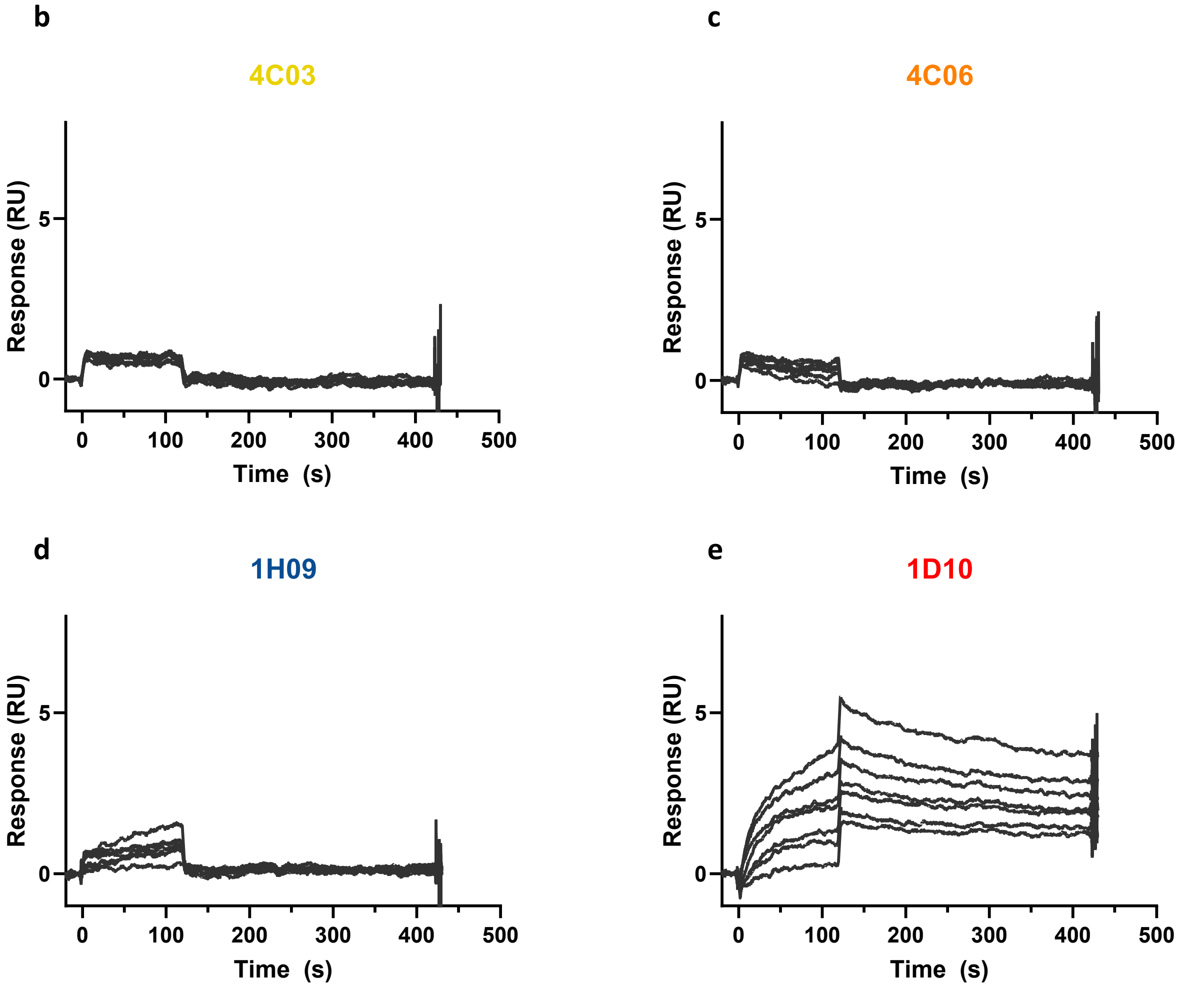

Supplementary Fig. 4: Epitope binning and binding of VHHs to RV3 postF. a Numeric response values for epitope binning of the 13 lead VHHs, related to Figure 2c. **(b-e)** SPR sensorgrams for binding of each representative VHH to RV3 postF. Source data are provided within the Source Data file.

a

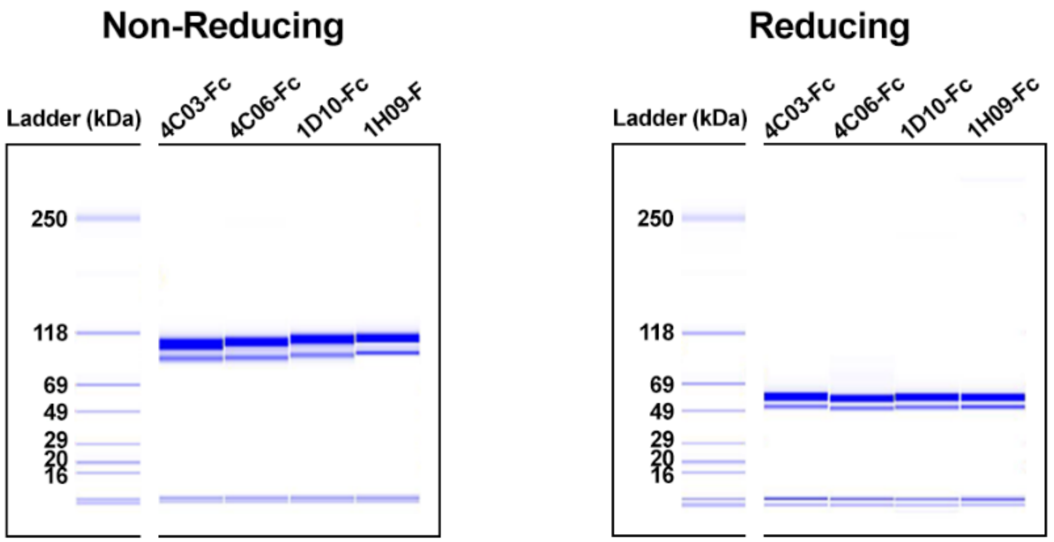

b

Neutralization of RV3-eGFP

VHH-Fc Name	4C03-Fc (*n*=2)	4C06-Fc (*n*=1)	1D10-Fc (*n*=2)	1H09-Fc (*n*=1)	PIA174 (*n*=1)
**IC_{50} (M)**	3.3E-11	1.2E-10	2.8E-11	4.6E-11	1.9E-10

Supplementary Fig. 5: Generation of VHH-Fc fusions and neutralization of RV3-eGFP. a Non-reducing and reducing SDS-PAGE gels for VHH-Fc fusion constructs showing purity and successful dimerization. Gels are cropped to exclude irrelevant lanes. **b** IC_{50} values for VHH-Fc constructs determined by neutralization assays performed with recombinant RV3-eGFP virus. PIA174 was included as a positive control antibody.

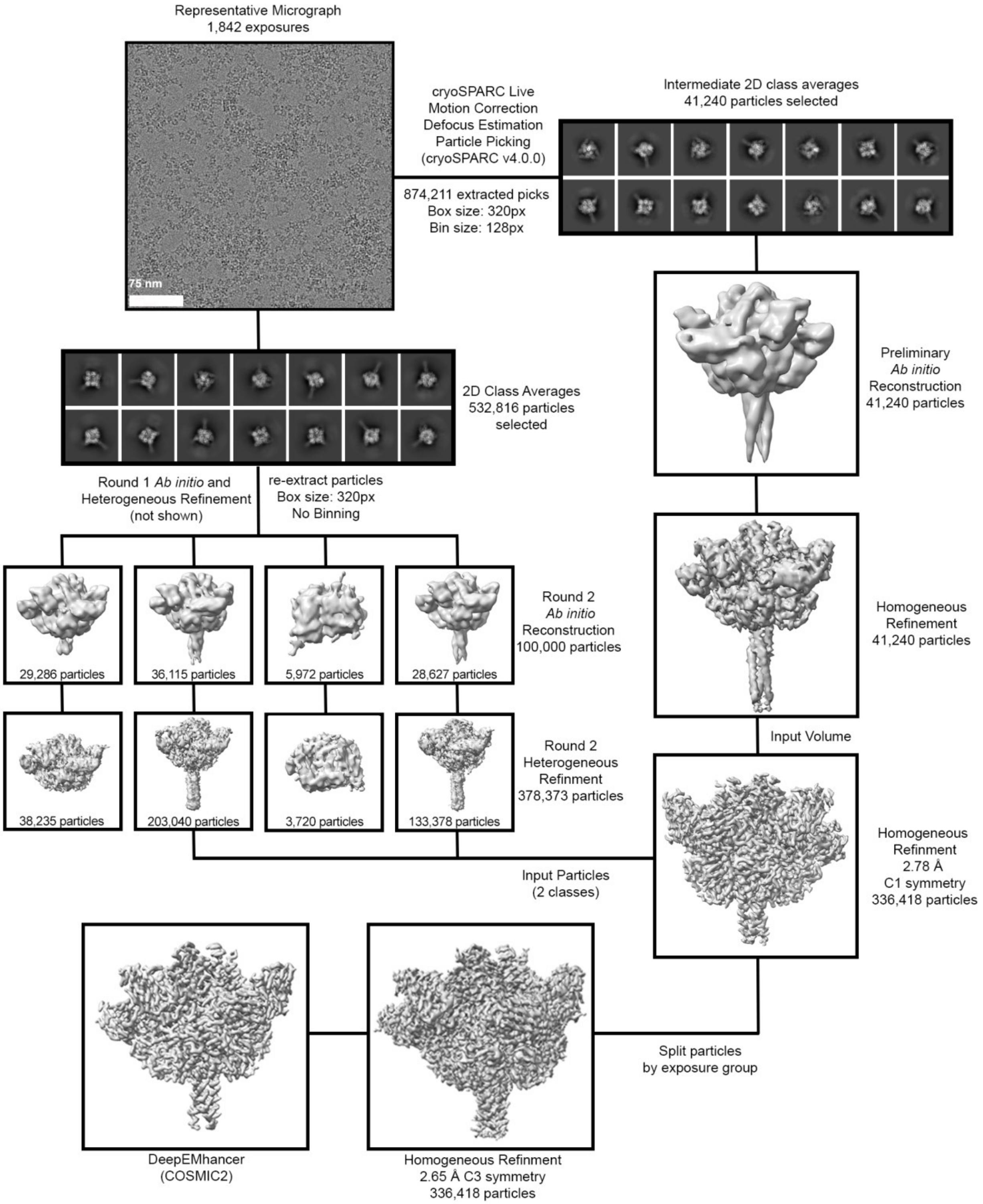

Supplementary Fig. 6: Cryo-EM data processing workflow for RV3 preF bound to VHHs 4C03 and 4C06.

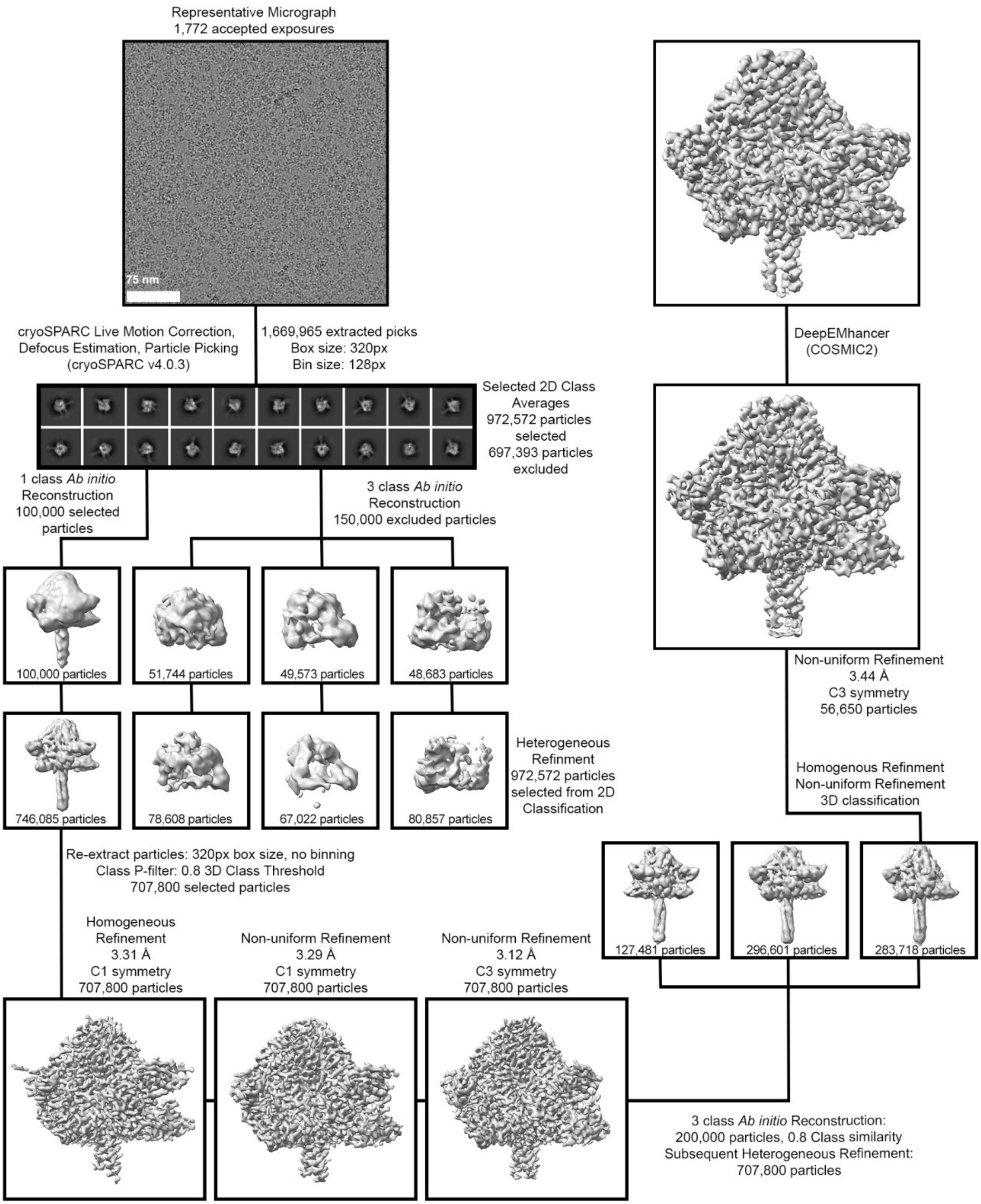

Supplementary Fig. 7: Cryo-EM data processing workflow for RV3 preF bound to VHHs 1D10 and 4C06.

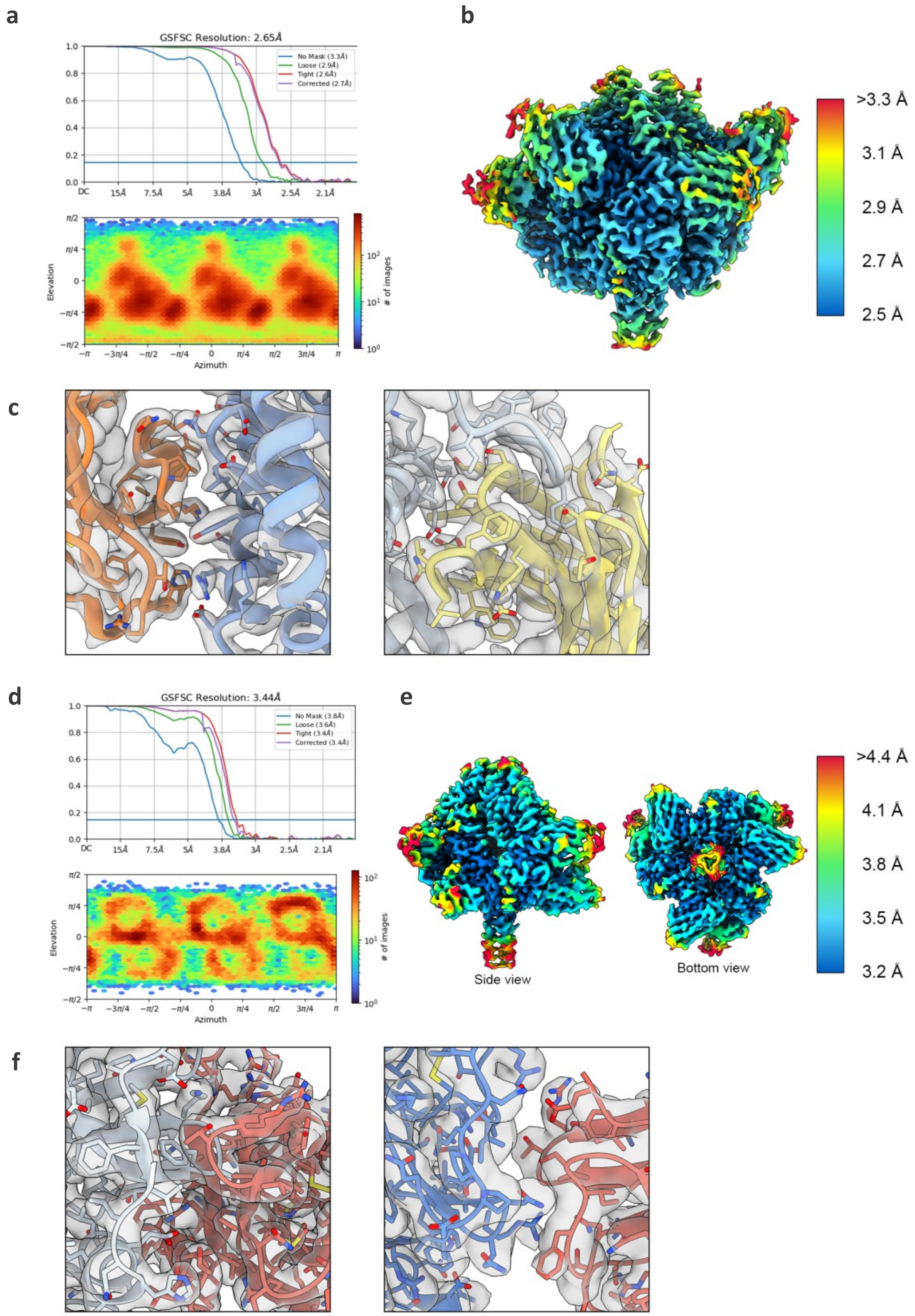

Supplementary Fig. 8: Cryo-EM structure validation. a Fourier shell correlation and viewing distribution plots for the RV3 preF + 4C03 + 4C06 final map. **b** Side view of RV3 preF + 4C03 + 4C06 final sharpened map, colored according to local resolution. **c** The binding interface for 4C03 (left) and 4C06 (right) with RV3 preF. Cryo-EM map is shown as a transparent surface with the built-in model shown as ribbons. F protomers are colored shades of blue, 4C03 is colored orange, and 4C06 is colored yellow. **d** Fourier shell correlation and viewing distribution plots for RV3 preF + 1D10 + 4C06 final map. **e** Side and bottom view of RV3 preF + 1D10 + 4C06 final sharpened map, colored according to local resolution. **f** Primary 1D10 binding interface (left) and contacts with the adjacent protomer (right) within RV3 preF. Cryo-EM map is shown as a transparent surface with the built-in model shown as ribbons. F protomers are colored shades of blue and 1D10 is colored red.

Supplementary Table 1: IC_{50} values for neutralization of RV3-eGFP

Clone ID	Geometric Mean IC_{50} (M) RV3-eGFP	Biological Replicates (*n*)
1A03	2.2E-10	3
1H09	1.9E-10	3
2B03	1.8E-07	2
2E10	2.9E-10	3
2H02	2.4E-10	3
3A02	1.5E-08	2
3E06	2.1E-08	2
4C03	2.1E-10	3
4C06	1.6E-10	3
4E07	1.5E-07	2
5D03	3.8E-08	2
5E03	5.3E-07	2
6B02	7.6E-08	2
6H03	2.2E-07	2
1D10	4.4E-10	4
3B04	1.4E-10	4
1A04	N.D.	-
1B07	N.D.	-
1E08	N.D.	-
3A03	N.D.	-
3B08	N.D.	-
3C02	N.D.	-
3E09	N.D.	-
4D03	N.D.	-
4F05	N.D.	-
4G04	N.D.	-
5B02	N.D.	-
5F02	N.D.	-
6A02	N.D.	-
6A06	N.D.	-
06B01	N.D.	-
6C05	N.D.	-
6G04	N.D.	-
6H10	N.D.	-
6E05	N.D.	-
1H11	N.D.	-
5B11	N.D.	-
5A04	N.D.	-
5F11	N.D.	-

N.D. = not determined

Supplementary Table 2: Amino acid sequences for 13 neutralizing VHHs

VHH	Amino Acid Sequence
1A03	EVQLVESGGGLVQPGGSLTLSCAVSGDISSINVMNWYRQAPGKRREFLAR<br>ITANGYRNYADSVEGRFTISRDDAAKNTVYLRMDRLEPEDTAVYSCKAEG<br>GWGDEFWGQGTQVTVSS
**1H09**	QVQLVESGGDLVQPGGSLRLSCGASGNIFEVARMDWHRQVPGKAREVVA<br>EIFAAGNTNYADHAKGRFTISRDAAENTVYLQMNGLRPEDTAAYFCSALIR<br>DNRGTWKEYWGPGTQVTVSS
2B03	EVQLVESGGGLVQAGGSLRLSCTSSESIYSYRVMAWHRQVPGKQRELVA<br>SITNSGAINYADFVKDRFTISRDNAKNTLLLQMNNLEPEDTAVYFCVAATTR<br>LYWGRGTQVTVSL
2E10	QVQLVESGGGLVQAGGSLKLSCAASGIILGDNAMAWYRRAPGKQRELVA<br>RITSAGSTDYTLSVEGRFTISRDNAKNTLYLQMNRLQFEDTAVYYCAALRS<br>WGENYWGQGTQVTVSS
2H02	QVQLVESGGGLVQAGGSLRLSCTASGSTPTIKTLAWYRQAPGKQRELVAE<br>IDVFGKTQYAPSVKGRFTISADNAKNTMYLEMNNLKPEDAAVYFGRASTFA<br>DNVYWGRGTQVTVSS
3A02	QVQLVESGGGLVQPGGSLRLSCAASTSISSIDAMGWYRQAPGKQREFVA<br>RITNRGITGHADSVKGRFTISGDNTKNTIYLQMNNLKYEDSAVYFCNAAHF<br>RGGVYWGQGTQVTVSS
3E06	QVQLVESGGGLVQPGGSLRLSCAVSGPIYSDYTMAWFRQAPGKEREFVA<br>RITWDGSTTNYADSVKGRFAISRDDAKKTAYLQMNSLTPDDTAVYTCAAAF<br>RSDRNNVWGQGTQVTVSS
**4C03**	EVQLVESGGGLVRAGGSLRLSCAASLRDLHTRTFYMGWFRQDPGKEREF<br>VAAIDWNTGAASYPDSVKGRFTISKDNARNAVYLQMNNLKPEDTAVYYCA<br>VGRPPLNRPTLAYYWGQGTQVTVSS
**4C06**	EVQLVESGGGLVQPGGSLRLSCSASGSLSTIKALGWYRRAPGRERELVAS<br>ITSAGETNYADSAKGRFTVSTDNAKNTVDLRMNSLKPEDTAVYYCYAESFV<br>LNIYFGQGTQVTVSS
4E07	EVQLVESGGGLVQAGGSLRLSCAASGSIFSISNMGWFRQAPGKEREFVG<br>RILWEGGETYYSDSVKGRFTISRDSAKTVMYLQMNNVKPEDTAIYYCTATR<br>PISNIYWGQGTQVTVSS
6H03	QVQLVESGGGLVQAGGSLKLSCAASGSSGTAKSMGWYRQTPGKQRELV<br>ARIFSDGSTNYEESVLGRFTISTDAAKNTMYLQMNSLKPEDTAVYYCYAEQ<br>FSLAIYWGQGTQVTVSS
**1D10**	EVQLVESGGGLVQTGDSLRLSCAASGSIFGENAMAWFRQAPGKQRELVA<br>RVSTGGTLFYADFAKVRFTISRDTAKQTVYLQMSSLRPEDTAVYYCAVAVG<br>TRNYWGQGTQVTVSS
3B04	QVQLVESGGGLVQPGGSLRLSCAASGSGGSVNNLNWYRQAPGKQREWV<br>AGISNIGILKYGDSMKGRATISSDNAKNTMYLQMSNLKPEDTAVYYCNYYT<br>PLTGDYWGQGTQVTVSS

Supplementary Table 3: EC_{50} values for 13 lead VHHs binding to pre- and postF

**Clone ID**	**EC_{50} (M) RV3 preF (*n*=1)**	**EC_{50} (M) RV3 postF (*n*=1)**
1A03	1.1E-10	-
**1H09**	1.5E-10	-
2B03	3.7E-09	-
2E10	1.2E-10	-
2H02	1.4E-10	-
3A02	2.8E-10	-
3E06	8.9E-10	-
**4C03**	8.0E-11	-
**4C06**	1.4E-10	-
4E07	3.0E-09	-
6H03	1.5E-10	-
**1D10**	4.8E-11	3.2E-08
3B04	2.2E-10	2.6E-08

Supplementary Table 4: Cryo-EM Data Collection, Reconstruction and Model Validation

**EM DATA COLLECTION**		
Microscope (FEI)	Talos Glacios	Talos Glacios
Voltage (kV)	200	200
Detector	Falcon 4	Falcon 4
Pixel size (Å/pix)	0.94	0.94
Exposure rate (e^-/pix/s)	3.6	3.6
Frames per exposure	60	60
Exposure (e^-/Å^2)	50	50
Defocus range (μm)	1.5-2.5	1.5-2.5
Tilt angle (°)	0	30
Micrographs collected	1,842	1,993
Micrographs used	1,534	1,772
Particles extracted	874,211	1,530,217
Automation software	SerialEM	SerialEM
**Complex Composition**	**RV3 F + 4C03 VHH + 4C06 VHH**	**RV3 F + 1D10 VHH + 4C06 VHH**
**3D RECONSTRUCTION STATISTICS**		
Particles	336,418	56,650
Symmetry	C3	C3
Map sharpening B-factor	-117.6	-127.4
Resolution (Å) at FSC (using sharpened map)		
Unmasked: 0.5	3.0	3.7
Masked: 0.5	2.9	3.6
Unmasked: 0.143	2.8	3.4
Masked: 0.143	2.6	3.4
**MODEL REFINEMENT AND VALIDATION STATISTICS**		
Composition		
Amino Acids (#)	1,980	1,980
Ligands (Type: #)	NAG: 3	
Bonds (RMSD)		
Length (Å) (# > 4s)	0.010 (0)	0.002 (0)
Angles (°) (# > 4s)	0.74 (0)	0.56 (0)
Ramachandran plot		
Outliers (%)	0	0
Allowed (%)	1.2	0.9
Favored (%)	98.8	99.1
Rotamer outliers (%)	0	0
C-β outliers (%)	0	0
CaBLAM outliers (%)	0.48	0.32
ADP (B-factors)		
Amino Acids (mean)	37.02	81.25
Ligand (mean)	60.41	
CC (mask)	0.74	0.78
MolProbity score	1.15	0.98
Clash score	3.61	2.06
EMRinger score	4.89	2.90
**EMDB ID**	42983	42987
**PDB ID**	8V5K	8V62

Supplementary Table 5: VHH-Fc Amino Acid Sequences

VHH-Fc	Amino Acid Sequence
4C03-Fc	EVQLVESGGGLVRAGGSLRLSCAASLRDLHTRTFYMGWFRQDPGKEREFVAAI<br>DWNTGAASYPDSVKGRFTISKDNARNAVYLQMNNLKPEDTAVYYCAVGRPPLN<br>RPTLAYYWGQGTQVTVSSDKTHTCPPCPAPELLGGPSVFLFPPKPKDTLMISRT<br>PEVTCVVVDVSHEDPEVKFNWYVDGVEVHNAKTKPREEQYNSTYRVVSVLTVL<br>HQDWLNGKEYKCKVSNKALPAPIEKTISKAKGQPREPQVYTLPPSREEMTKNQV<br>SLTCLVKGFYPSDIAVEWESNGQPENNYKTTPPVLDSDGSFFLYSKLTVDKSRW<br>QQGNVFSCSVMHEALHNHYTQKSLSLSPGK
4C06-Fc	EVQLVESGGGLVQPGGSLRLSCSASGSLSTIKALGWYRRAPGRERELVASITSA<br>GETNYADSAKGRFTVSTDNAKNTVDLRMNSLKPEDTAVYYCYAESFVLNIYFGQ<br>GTQVTVSSDKTHTCPPCPAPELLGGPSVFLFPPKPKDTLMISRTPEVTCVVVDVS<br>HEDPEVKFNWYVDGVEVHNAKTKPREEQYNSTYRVVSVLTVLHQDWLNGKEYK<br>CKVSNKALPAPIEKTISKAKGQPREPQVYTLPPSREEMTKNQVSLTCLVKGFYPS<br>DIAVEWESNGQPENNYKTTPPVLDSDGSFFLYSKLTVDKSRWQQGNVFSCSVM<br>HEALHNHYTQKSLSLSPGK
1H09-Fc	QVQLVESGGDLVQPGGSLRLSCGASGNIFEVARMDWHRQVPGKAREVVAEIFA<br>AGNTNYADHAKGRFTISRDAAENTVYLQMNGLRPEDTAAYFCSALIRDNRGTWK<br>EYWGPGTQVTVSSDKTHTCPPCPAPELLGGPSVFLFPPKPKDTLMISRTPEVTC<br>VVVDVSHEDPEVKFNWYVDGVEVHNAKTKPREEQYNSTYRVVSVLTVLHQDWL<br>NGKEYKCKVSNKALPAPIEKTISKAKGQPREPQVYTLPPSREEMTKNQVSLTCLV<br>KGFYPSDIAVEWESNGQPENNYKTTPPVLDSDGSFFLYSKLTVDKSRWQQGNV<br>FSCSVMHEALHNHYTQKSLSLSPGK
1D10-Fc	EVQLVESGGGLVQTGDSLRLSCAASGSIFGENAMAWFRQAPGKQRELVARVST<br>GGTLFYADFAKVRFTISRDTAKQTVYLQMSSLRPEDTAVYYCAVAVGTRNYWGQ<br>GTQVTVSSDKTHTCPPCPAPELLGGPSVFLFPPKPKDTLMISRTPEVTCVVVDVS<br>HEDPEVKFNWYVDGVEVHNAKTKPREEQYNSTYRVVSVLTVLHQDWLNGKEYK<br>CKVSNKALPAPIEKTISKAKGQPREPQVYTLPPSREEMTKNQVSLTCLVKGFYPS<br>DIAVEWESNGQPENNYKTTPPVLDSDGSFFLYSKLTVDKSRWQQGNVFSCSVM<br>HEALHNHYTQKSLSLSPGK

Uncropped gel, Supplementary Figure S5a: Dimerization analysis of purified VHH-Fc constructs

3	SDP196013	(NR)	9	SDP196013	(R)
4	SDP196014	(NR)	10	SDP196014	(R)
5	SDP196015	(NR)	11	SDP196015	(R)
6	SDP196016	(NR)	12	SDP196016	(R)

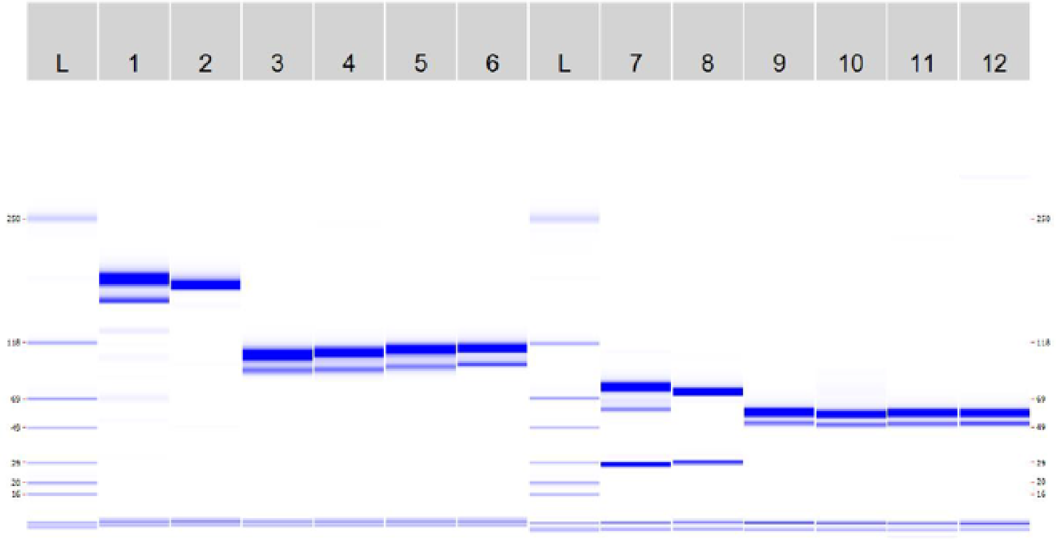

	Lane	Sample ID	VHH-Fc Name
	1	unrelated sample	
	2	unrelated sample	
Non-reducing SDS PAGE	3	SDP196013	4C03-Fc
	4	SDP196014	4C06-Fc
	5	SDP196015	1D10-Fc
	6	SDP196016	1H09-Fc
	7	unrelated sample	
	8	unrelated sample	
Reducing SDS PAGE	9	SDP196013	4C03-Fc
	10	SDP196014	4C06-Fc
	11	SDP196015	1D10-Fc
	12	SDP196016	1H09-Fc
