# Supplementary material for: Structural basis for potent neutralization of human respirovirus type 3 by protective single-domain camelid antibodies
Source: Nat Commun. 2024 Jun 27;15:5458. doi: 10.1038/s41467-024-49757-1 (PMC11211449; doi:10.1038/s41467-024-49757-1)
Supplement: Supplementary file 3 — Reporting Summary [file 41467_2024_49757_MOESM3_ESM.pdf]

nature portfolio

Corresponding author(s): Jason S. McLellan

Last updated by author(s): May 9, 2024

Reporting Summary

Nature Portfolio wishes to improve the reproducibility of the work that we publish. This form provides structure for consistency and transparency in reporting. For further information on Nature Portfolio policies, see our Editorial Policies and the Editorial Policy Checklist.

Statistics

For all statistical analyses, confirm that the following items are present in the figure legend, table legend, main text, or Methods section.

n/a	Confirmed	
☐	☒	The exact sample size (*n*) for each experimental group/condition, given as a discrete number and unit of measurement
☐	☒	A statement on whether measurements were taken from distinct samples or whether the same sample was measured repeatedly
☐	☒	The statistical test(s) used AND whether they are one- or two-sided<br>*Only common tests should be described solely by name; describe more complex techniques in the Methods section.*
☒	☐	A description of all covariates tested
☐	☒	A description of any assumptions or corrections, such as tests of normality and adjustment for multiple comparisons
☐	☒	A full description of the statistical parameters including central tendency (e.g. means) or other basic estimates (e.g. regression coefficient) AND variation (e.g. standard deviation) or associated estimates of uncertainty (e.g. confidence intervals)
☐	☒	For null hypothesis testing, the test statistic (e.g. *F*, *t*, *r*) with confidence intervals, effect sizes, degrees of freedom and *P* value noted<br>*Give P values as exact values whenever suitable.*
☒	☐	For Bayesian analysis, information on the choice of priors and Markov chain Monte Carlo settings
☒	☐	For hierarchical and complex designs, identification of the appropriate level for tests and full reporting of outcomes
☒	☐	Estimates of effect sizes (e.g. Cohen's *d*, Pearson's *r*), indicating how they were calculated

Our web collection on statistics for biologists contains articles on many of the points above.

Software and code

Policy information about availability of computer code

Data collection	Serial EM v4.0.10, BIACORE T200 Acquisition Software, Octet RED HTX Data Acquisition Software,
Data analysis	PHENIX v1.18.2, Coot v0.9.2, GraphPad Prism v10.2.1, cryoSparc v4.0.0 and v4.0.3, ChimeraX v1.7, Isolde v1.7, Forte´Bio software Octet Data Analysis High Throughput v10.0, BIACORE T200 Evaluation Software

For manuscripts utilizing custom algorithms or software that are central to the research but not yet described in published literature, software must be made available to editors and reviewers. We strongly encourage code deposition in a community repository (e.g. GitHub). See the Nature Portfolio guidelines for submitting code & software for further information.

Data

Policy information about availability of data

All manuscripts must include a data availability statement. This statement should provide the following information, where applicable:

- Accession codes, unique identifiers, or web links for publicly available datasets
- A description of any restrictions on data availability
- For clinical datasets or third party data, please ensure that the statement adheres to our policy

The amino acid sequences of the 13 neutralizing VHHs are available in Supplementary Table 2 and the sequences for the VHH-Fc constructs are in Supplementary Table 5. Structural models are deposited in the protein data bank (PDB, https://www.rcsb.org/). The PDB IDs are: 8V5K and 8V62. Cryo-EM maps are deposited in the EM database (https://www.emdataresource.org/). The maps are publicly available and can be found using the following EMD IDs: 42983, 42987. All data supporting the findings of this study are within the article and its Supplementary Information. Source data are provided with this paper.

Research involving human participants, their data, or biological material

Policy information about studies with human participants or human data. See also policy information about sex, gender (identity/presentation), and sexual orientation and race, ethnicity and racism.

Reporting on sex and gender	*Use the terms sex (biological attribute) and gender (shaped by social and cultural circumstances) carefully in order to avoid confusing both terms. Indicate if findings apply to only one sex or gender; describe whether sex and gender were considered in study design; whether sex and/or gender was determined based on self-reporting or assigned and methods used.*<br>*Provide in the source data disaggregated sex and gender data, where this information has been collected, and if consent has been obtained for sharing of individual-level data; provide overall numbers in this Reporting Summary. Please state if this information has not been collected.*<br>*Report sex- and gender-based analyses where performed, justify reasons for lack of sex- and gender-based analysis.*
Reporting on race, ethnicity, or other socially relevant groupings	*Please specify the socially constructed or socially relevant categorization variable(s) used in your manuscript and explain why they were used. Please note that such variables should not be used as proxies for other socially constructed/relevant variables (for example, race or ethnicity should not be used as a proxy for socioeconomic status).*<br>*Provide clear definitions of the relevant terms used, how they were provided (by the participants/respondents, the researchers, or third parties), and the method(s) used to classify people into the different categories (e.g. self-report, census or administrative data, social media data, etc.)*<br>*Please provide details about how you controlled for confounding variables in your analyses.*
Population characteristics	*Describe the covariate-relevant population characteristics of the human research participants (e.g. age, genotypic information, past and current diagnosis and treatment categories). If you filled out the behavioural & social sciences study design questions and have nothing to add here, write "See above."*
Recruitment	*Describe how participants were recruited. Outline any potential self-selection bias or other biases that may be present and how these are likely to impact results.*
Ethics oversight	*Identify the organization(s) that approved the study protocol.*

Note that full information on the approval of the study protocol must also be provided in the manuscript.

Field-specific reporting

Please select the one below that is the best fit for your research. If you are not sure, read the appropriate sections before making your selection.

☒ Life sciences ☐ Behavioural & social sciences ☐ Ecological, evolutionary & environmental sciences

For a reference copy of the document with all sections, see nature.com/documents/nr-reporting-summary-flat.pdf

Life sciences study design

All studies must disclose on these points even when the disclosure is negative.

Sample size	Sample sizes were chosen to minimize the number of cotton rats used in the study while providing sufficient statistical power to observe significant differences.
Data exclusions	No data were excluded.
Replication	We did not attempt to replicate the cotton rat prophylactic protection studies due to limited resources.
Randomization	Coton rats were randomly assigned to experimental groups
Blinding	Investigators were blinded to group allocation during data collection.

Reporting for specific materials, systems and methods

We require information from authors about some types of materials, experimental systems and methods used in many studies. Here, indicate whether each material, system or method listed is relevant to your study. If you are not sure if a list item applies to your research, read the appropriate section before selecting a response.

Materials & experimental systems

n/a	Involved in the study
☐	☒ Antibodies
☐	☒ Eukaryotic cell lines
☒	☐ Palaeontology and archaeology
☐	☒ Animals and other organisms
☒	☐ Clinical data
☒	☐ Dual use research of concern
☒	☐ Plants

Methods

n/a	Involved in the study
☒	☐ ChIP-seq
☒	☐ Flow cytometry
☒	☐ MRI-based neuroimaging

Antibodies

Antibodies used	Antibody PIA174, 15 mg/kg for animal studies and 500 nM concentration for BLI evaluation of the RV3 preF antigen.
Validation	Stewart-Jones, G.B.E., et al., Structure-based design of a quadrivalent fusion glycoprotein vaccine for human parainfluenza virus types 1-4. Proc Natl Acad Sci U S A, 2018. 115(48): p. 12265-12270. (SI Appendix, Fig S1 and Table S1)

Eukaryotic cell lines

Policy information about cell lines and Sex and Gender in Research

Cell line source(s)	Expi293F cells (Gibco, Catalog number: A14527), LLC-MK2 cells (ATCC, CCL-7)
Authentication	Cell lines were purchased commercially and were not further validated.
Mycoplasma contamination	Confirmed that all cell lines are free of Mycoplasma by PCR
Commonly misidentified lines (See ICLAC register)	No commonly misidentified cell lines were used in this study.

Animals and other research organisms

Policy information about studies involving animals; ARRIVE guidelines recommended for reporting animal research, and Sex and Gender in Research

Laboratory animals	Llama glama (~ 1 year old), Sigmodon hispidus (6-8 weeks old)
Wild animals	*Provide details on animals observed in or captured in the field; report species and age where possible. Describe how animals were caught and transported and what happened to captive animals after the study (if killed, explain why and describe method; if released, say where and when) OR state that the study did not involve wild animals.*
Reporting on sex	all male
Field-collected samples	*For laboratory work with field-collected samples, describe all relevant parameters such as housing, maintenance, temperature, photoperiod and end-of-experiment protocol OR state that the study did not involve samples collected from the field.*
Ethics oversight	Llama immunization studies were performed under approval by the Research Ethics Committee (CER, protocol #CE-SANTE-001). Approximately one-year-old male llamas (N=3) were used for the studies. Cotton rat studies were conducted under applicable laws and guidelines and after approval from Sigmovir Biosystems, Inc.'s Institutional Animal Care and Use Committee (IACUC). Inbred S. hispidus cotton rats were obtained from a colony maintained at Sigmovir Biosystems, Inc. Six to eight-week-old male cotton rats (N=42) were used for the studies. Animals were housed in large polycarbonate cages and fed a standard diet of rodent chow and water ad libitum.

Note that full information on the approval of the study protocol must also be provided in the manuscript.
